# Performance of capnometry in non-intubated infants in the pediatric intensive care unit

**DOI:** 10.1186/1471-2431-14-163

**Published:** 2014-06-25

**Authors:** Bria M Coates, Robin Chaize, Denise M Goodman, Ranna A Rozenfeld

**Affiliations:** 1 Division of Critical Care, Northwestern University Feinberg School of Medicine and Ann & Robert H. Lurie Children’s Hospital of Chicago, 225 E. Chicago Ave, Box 73, Chicago, Illinois 60611, USA; 2 Joe DiMaggio Children’s Hospital, 1005 Joe DiMaggio Drive Hollywood, Florida 33021, USA

**Keywords:** Capnometry, Ventilation, Monitoring, Infants, Microstream, Carbon dioxide

## Abstract

**Background:**

Assessing the ventilatory status of non-intubated infants in the Pediatric Intensive Care Unit (PICU) is a constant challenge. Methods to evaluate ventilation include arterial blood gas analysis (ABG), which is invasive and intermittent, and transcutaneous carbon dioxide monitoring (P_tcCO2_), which, while non-invasive, is also intermittent. A method that is non-invasive and continuous would be of great benefit in this population. We hypothesized that non-invasive capnometry via sidestream monitoring of exhaled carbon dioxide (CO_2_) would provide an acceptable measurement of ventilatory status when compared to ABG or P_tcCO2_.

**Methods:**

Preliminary prospective study of infants less than one year of age admitted to the PICU in a large urban teaching hospital. Infants not intubated and not requiring non-invasive ventilation were eligible. A sidestream CO_2_ reading was obtained in a convenience sample of 39 patients. A simultaneous ABG was collected in those with an arterial catheter, and a P_tcCO2_ was obtained in those without.

**Results:**

Correlation of sidestream CO_2_ with ABG was excellent (r^2^ = 0.907). Sidestream correlated less well with P_tcCO2_ (r^2^ = 0.649). Results were not significantly altered when weight and respiratory rate were added as independent variables. Bland-Altman analysis revealed a bias of -2.7 with a precision of ±6.5 when comparing sidestream CO_2_ to ABG, and a bias of -1.7 with a precision of ±9.9 when comparing sidestream CO_2_ to P_tcCO2_.

**Conclusions:**

Performance of sidestream monitoring of exhaled CO_2_ is acceptable clinical trending to assess the effectiveness of ventilation in non-intubated infants in the PICU.

## Background

Respiratory monitoring of non-intubated children in the pediatric intensive care unit (PICU) is a constant challenge. Effectiveness of the patient’s respiratory effort can be monitored, to some extent, by visual observation of chest expansion, rate and depth of respirations, use of accessory muscles, and the quality and quantity of breath sounds. These subjective findings can be misleading, however, and objective means of following oxygenation and ventilation are needed in the majority of PICU patients. Pulse oximetry has become the standard of care for oxygenation monitoring in both the PICU and the general ward [1]. Monitoring of ventilation poses a more difficult challenge. Except in extreme cases, pulse oximetry will not reliably detect hypoventilation. Measurement of the partial pressure of carbon dioxide (P_aCO2_) through arterial blood gas analysis (ABG) is the gold standard for assessing ventilation. While a true reflection of ventilation, measurement of the P_aCO2_ is both invasive and intermittent, which greatly limits its use. Consequently, other measures of ventilatory monitoring have been established.

Transcutaneous carbon dioxide monitoring (P_tcCO2_) estimates the P_aCO2_ by warming the skin to induce hyperperfusion, enabling the electrochemical measurement of the partial pressure of oxygen and carbon dioxide [2]. Transcutaneous monitoring is considered a safe procedure, however tissue injury may occur at the measuring site, including blisters, burns, and skin tears. These complications are rare with current technology, and primarily occur when the P_tcCO2_ is left in place for long periods of time, so continuous monitoring is generally avoided. In patients with poor skin integrity or adhesive allergy, transcutaneous monitoring may be relatively contraindicated [3,4]. Various clinical factors may increase the discrepancy between arterial and transcutaneous values of carbon dioxide, including hyperoxia (P_aO2_ > 100 torr), hypoperfusion, improper electrode placement or application, body wall edema, and the thickness of the patient’s skin or subcutaneous tissue [3-5].

Capnography is regularly used in operating rooms and intensive care units to monitor carbon dioxide clearance in tracheally intubated patients [6-8]. Exhaled carbon dioxide generally reflects P_aCO2_, but the correlation decreases predictably with increasing dead space ventilation [9]. In recent years, there has been widening use of oral and nasal capnometry for monitoring ventilation in non-intubated adults and children [10,11]. Noninvasive capnography has been used in emergency departments, pediatric intensive care units, during polysomnography, during sedation, and during interfacility transport [10-18]. However, the ability of these devices to reliably capture exhaled CO_2_ from non-intubated infants with high respiratory rates and low tidal volumes is unknown [19]. We prospectively compared sidestream carbon dioxide (CO_2_), with P_tcCO2_ and/or P_aCO2_ in infants less than one year of age admitted to the PICU to determine if sidestream monitoring provides an acceptable measurement of the effectiveness of ventilation. Should the performance of this technology prove acceptable for clinical trending, it would provide the benefits of both non-invasive and continuous monitoring of ventilatory status.

## Methods

This study was approved by the Institutional Review Board at Children’s Memorial Hospital in Chicago, Illinois, now the Ann & Robert H. Lurie Children’s Hospital of Chicago. Infants admitted to the PICU who were age 1 year or less, without a tracheostomy or immediate need for invasive or non-invasive ventilation were eligible for enrollment. Additional information was recorded on each subject including age in months, weight in kilograms (kg), respiratory rate, and diagnosis. A convenience sample of subjects was enrolled from March 2007 – September 2008.

Sidestream sampling was performed on all subjects in the study. The sidestream cannula (Philips Microstream EtCo2 circuit, Smart Capnoline O_2_), a two prong nasal cannula with a CO_2_ detection port that hangs in front of the mouth, was placed on the subject and left in place until a steady state reading was obtained. The sampling rate is 50 ml/min and the sampling line is 100 cm long. Oxygen can be delivered through the nasal prongs, if desired, and was only used as indicated for patient care. The value of the exhaled CO_2_ was recorded (Microcap Microstream Oridion Machine) after a consistent reading had been present for 2 minutes. For those subjects without an arterial catheter in place, a P_tcCO2_ (Radiometer Copenhagen, Tina TCM 4) was obtained. The P_tcCO2_ machine was calibrated prior to placing the sidestream cannula on the subject. The P_tcCO2_ electrode was placed at the same time as the sidestream nasal cannula and the reading was recorded when no further increase was observed for 30-60 seconds. In those subjects who had an indwelling arterial catheter in place as part of their routine care, an arterial blood gas was drawn immediately after the sidestream reading was recorded. The majority of subjects had a single sidestream CO_2_ measurement and either a P_tcCO2_ or a P_aCO2_, but 4 subjects had both a P_tcCO2_ and a P_aCO2_.

The correlation between a single sidestream CO_2_ reading and the simultaneous P_tcCO2_ or P_aCO2_ was examined using Spearman’s rho. Since correlation is expected when two methods attempt to measure the same physiologic parameter, a Bland Altman analysis was also conducted [20] to analyze the differences between the sidestream reading and either the P_tcCO2_ or the P_aCO2_. Bias, the mean difference between values, and precision, the standard deviation (SD) of the bias, were calculated for P_tcCO2_ to sidestream and P_aCO2_ to sidestream differences.

The effect of respiratory rate and weight on the relationship between P_tcCO2_ or P_aCO2_ and sidestream CO_2_ was assessed by simple linear regression models for the unadjusted effect and multiple linear regression models for the adjusted effect. We examined both absolute values of sidestream CO_2_ and log-transformed sidestream due to the non-normal distribution of the sidestream values.

## Results

Forty-three sample sets were obtained from 39 subjects for analysis. Please see Table 1 for full demographic data. In the PtcCO_2_ comparison group there were 29 subjects. Admission diagnoses included respiratory illness (n = 16), cardiothoracic surgery (n = 9), and other (n = 4). In the ABG comparison group there were 14 subjects. Admission diagnoses included respiratory illness (n = 1), cardiothoracic surgery (n = 12), and other (n = 1). The predominance of cardiothoracic surgery subjects in the ABG group reflects the practice patterns at our institution for placing and keeping arterial lines in non-intubated subjects (Table 1).

**Table 1:** Demographics

	**PtcCO**_**2**_	**ABG**
Number of patients	29	14
Age (months)	3.4 (2.8, 0.5-11)	4.1 (3.3, 0.3-11)
Weight (kg)	5.3 (1.8, 3-9.4)	5.3 (1.3, 3-7.1)
Diagnosis: respiratory	16	1
Cardiac	9	12
Other	4	1
Respiratory rate (breaths/min)	44 (15, 25-82)	45 (12, 32-69)

Results expressed as mean (SD, range).

**Figure 1:**
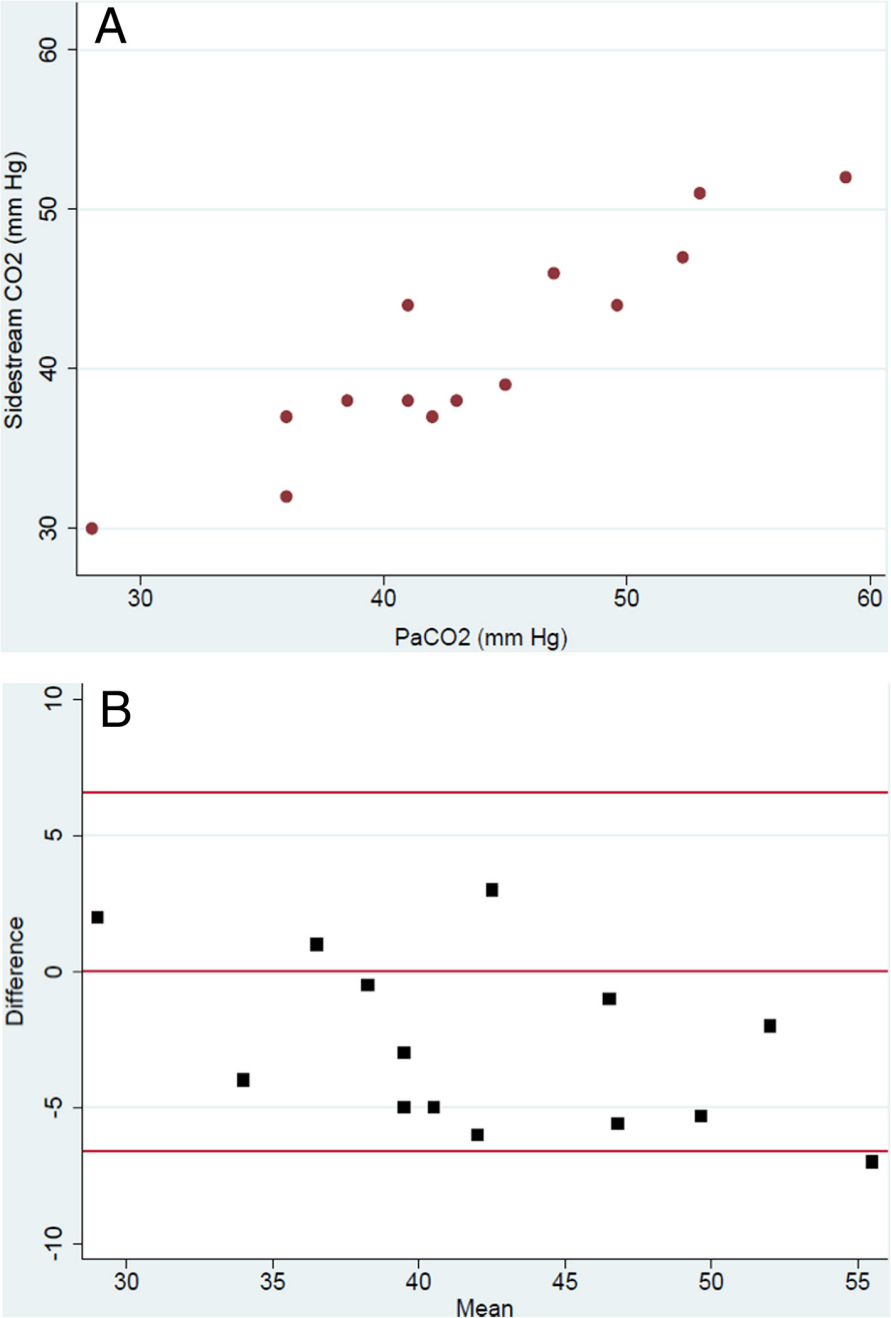
**Comparison of sidestream CO**_**2 **_**to PaCO**_**2**_**. A**: Correlation between sidestream CO_2_ and PaCO_2_ values (r^2^ = 0.907). **B**: Bland-Altman analysis of difference in CO_2_ between sidestream and PaCO_2_ (y-axis) versus average of measured sidestream and PaCO_2_ (x-axis), bias -2.7, precision ± 6.5.

**Figure 2:**
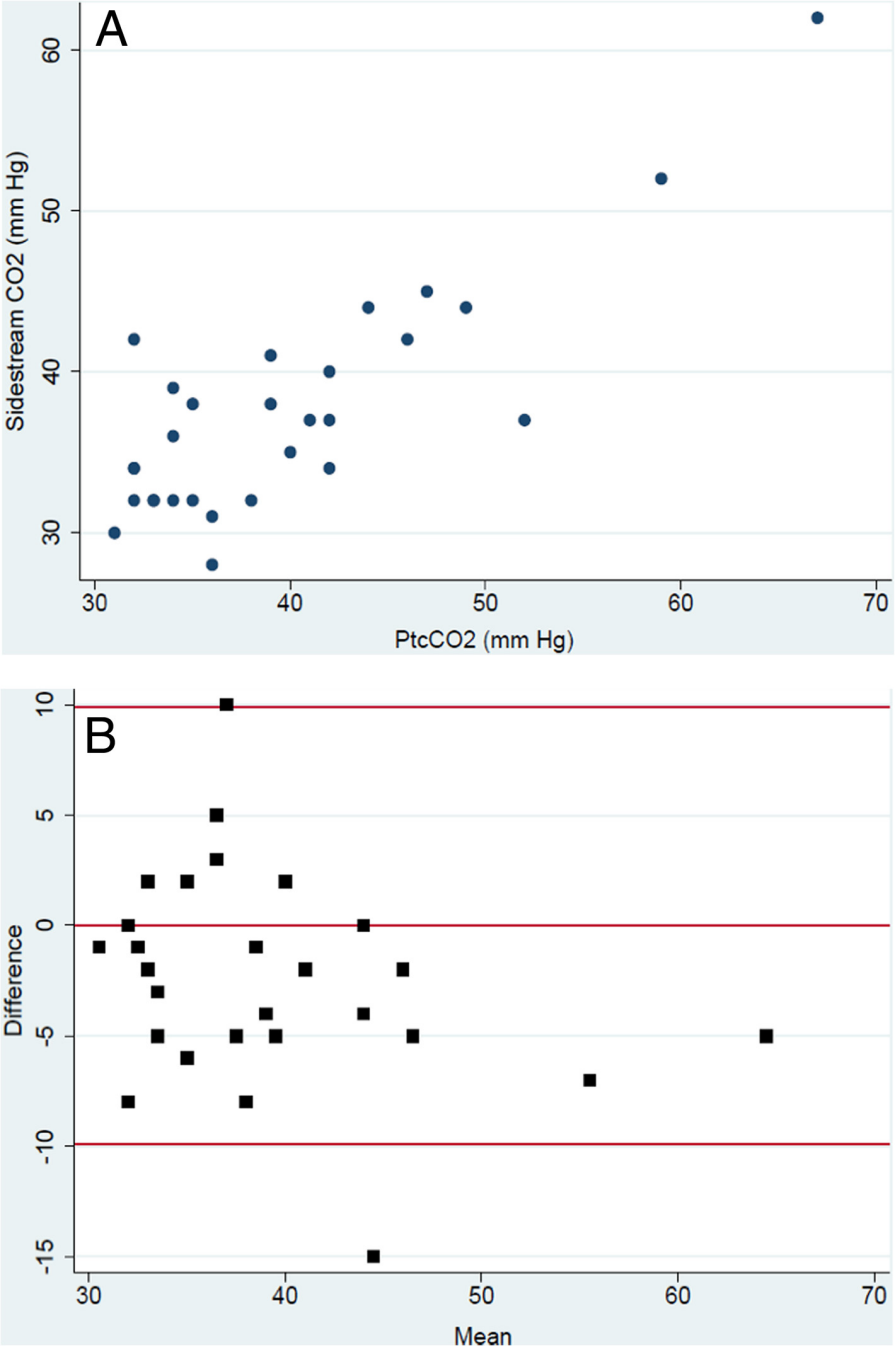
**Comparison of sidestream CO**_**2 **_**to PtcCO**_**2**_**. A**: Correlation between sidestream CO_2_ and PtcCO_2_ values (r^2^ = 0.649). **B**: Bland-Altman analysis of difference in CO_2_ between sidestream and PtcCO_2_ (y-axis) versus average of measured sidestream and PtcCO_2_ (x-axis), bias -1.7, precision ±9.9.

The correlation between sidestream CO_2_ and P_aCO2_ was excellent (r^2^ 0.907, Figure 1A). Sidestream CO_2_ correlated less well with P_tcCO2_ values (r^2^ 0.649, Figure 2A). The Bland-Altman analysis revealed good agreement between P_aCO2_ and sidestream CO_2_ (bias –2.7, precision ±6.5, Figure 1B). Agreement was less robust when comparing sidestream CO_2_ and PtcCO_2_ (bias –1.7, precision ±9.9, Figure 2B).

The influence of respiratory rate and weight on performance of sidestream CO_2_ monitoring is presented in Table 2. Sidestream performance was unaffected by either factor. Therefore, sidestream measurements were accurate across the range of size and respiratory rates found in the infants in this study.

**Table 2:** **Linear regression (dependent variable: log transformed sidestream CO**_**2**_**)**

**Independent variables**	**β (Unstandardized coefficient)**	**R**^**2**^	***p *** **value**
	Unadjusted effect		
Respiratory rate	-.003	.043	.195
Weight	-.019	.033	.248
PtcCO_2_	.016	.650	<.001
PaCO_2_	.019	.855	<.001
	Adjusted model, R^2^ = .668		
Respiratory rate	-.002		.266
Weight	.001		.965
PtcCO_2_	.016		<.001
	Adjusted model, R^2^ = .866		
Respiratory rate	.002		.395
Weight	.001		.936
PaCO_2_	.019		<.001

## Discussion

This study demonstrates that sidestream CO_2_ monitoring can provide an acceptable estimation of P_aCO2_ in infants with varying degrees of tachypnea_._ It is comparable to another commonly used non-invasive monitor, the transcutaneous CO_2_. To our knowledge, this is the only study comparing these different techniques in this age range. One retrospective study compared end tidal CO_2_ with venous CO_2_ and found them to be highly correlated [21]. This study included children age 5.5 months-20 years with a median age of 5.7 years. Additional studies in infants have examined the difference between sidestream CO_2_ and capillary CO_2_, or used exhaled mainstream CO_2_ to analyze the capnographic indices associated with bronchopulmonary dysplasia (BPD) [22,23]. One of the studies was consistent with our findings, showing good correlation between sidestream CO_2_ and capillary CO_2_ in preterm infants without lung disease [22]. However, the correlation between sidestream CO_2_ and capillary CO_2_ was lost in infants with BPD. The gradient between the sidestream and capillary CO_2_ values in the BPD group was attributed to dead space ventilation and the ventilation-perfusion mismatch characteristic of this lung disease. None of these studies specifically evaluated the performance of sidestream CO_2_ in comparison to PtcCO_2_ or P_aCO2_ in infants.

In most cases, an estimated CO_2_ that is within 5 mm Hg of the P_aCO2_ would be considered acceptable for making clinical decisions when an arterial value is unavailable. In this study, the majority of measurements were within this range. Our results contrast with an adult study that did not show good correlation between sidestream CO_2_ and P_aCO2_ in non-intubated patients [24]. These adults were very tachypneic, and the authors speculated that this was a reflection of abnormal lung function, leading them to conclude that the usefulness of sidestream monitoring was limited in the presence of lung disease. As with all end-tidal CO_2_ monitoring, it is important to note that dead space ventilation will increase the discrepancy between exhaled CO_2_ and P_aCO2_ or P_tcCO2_[9]. This may explain the gradients seen between sidestream CO_2_ and P_aCO2_ in this adult study, and sidestream CO_2_ and capillary CO_2_ in the study of premature infants with BPD mentioned above. However, in our population, sidestream CO_2_ closely approximated P_aCO2_, and to a slightly lesser degree, P_tcCO2_, over a wide range of respiratory rates and degrees of distress in these infants, suggesting that sidestream monitoring of exhaled CO_2_ could be quite useful in this population.

Accurate sidestream monitoring is advantageous over ABG or P_tcCO2_ as it provides both a continuous and non-invasive means of monitoring ventilation. It is also much less labor intensive when compared to the alternate methods. Effective transcutaneous monitoring is dependent on both technical and patient factors. Repetitive ABG analysis requires the presence of an indwelling arterial catheter which can be difficult, painful, and time consuming to place.

The primary limitation to this preliminary study was its small sample size. Larger numbers and repeated measurements would enable further elucidation of patient or operator characteristics that impact the precision and reliability of these measurements. As the gold standard, P_aCO2_ should still be obtained when either P_tcCO2_ or sidestream readings seem to contradict clinical assessment.

## Conclusion

Performance of sidestream monitoring of exhaled CO_2_ is acceptable for assessing the effectiveness of ventilation in non-intubated infants in the PICU. It should be considered when continuous monitoring of ventilation is desired to aid in the early detection of changes in clinical status.

## Abbreviations

ABG: Arterial blood gas; BPD: Bronchopulmonary dysplasia; CO_2_: Carbon dioxide; kg: Kilograms; PaCO_2_: Arterial partial pressure of carbon dioxide; PICU: Pediatric intensive care unit; PtcCO_2_: Transcutaneous carbon dioxide monitoring; SD: Standard deviation.

## Competing interests

The authors declare that they have no competing interests.

## Authors’ contributions

BMC recruited subjects and collected data for the study, drafted the initial manuscript, and approved the final manuscript as submitted. RC developed initial study design, recruited subjects and collected data for the study, and approved the final manuscript as submitted. DMG helped with initial conceptualization of the study, performed the statistical analysis, and approved the final manuscript as submitted. RAR developed final study design, recruited subjects and collected data for the study, revised the manuscript, and approved the final manuscript as submitted.

## Pre-publication history

The pre-publication history for this paper can be accessed here:

http://www.biomedcentral.com/1471-2431/14/163/prepub
